# Tailoring subunit vaccine immunogenicity: Maximizing antibody and T cell responses by using combinations of adenovirus, poxvirus and protein-adjuvant vaccines against *Plasmodium falciparum* MSP1^☆^

**DOI:** 10.1016/j.vaccine.2010.08.068

**Published:** 2010-10-18

**Authors:** Alexander D. Douglas, Simone C. de Cassan, Matthew D.J. Dicks, Sarah C. Gilbert, Adrian V.S. Hill, Simon J. Draper

**Affiliations:** Jenner Institute, Oxford University, UK

**Keywords:** Malaria, Viral vectored vaccine, MSP1, Adenovirus, Poxvirus, MVA, Protein vaccine, Adjuvant, Montanide, *Plasmodium falciparum*

## Abstract

Subunit vaccination modalities tend to induce particular immune effector responses. Viral vectors are well known for their ability to induce strong T cell responses, while protein-adjuvant vaccines have been used primarily for induction of antibody responses. Here, we demonstrate in mice using a *Plasmodium falciparum* merozoite surface protein 1 (PfMSP1) antigen that novel regimes combining adenovirus and poxvirus vectored vaccines with protein antigen in Montanide ISA720 adjuvant can achieve simultaneous antibody and T cell responses which equal, or in some cases surpass, the best immune responses achieved by either the viral vectors or the protein vaccine alone. Such broad responses can be achieved either using three-stage vaccination protocols, or with an equally effective two-stage protocol in which viral vectors are admixed with protein and adjuvant, and were apparent despite the use of a protein antigen that represented only a portion of the viral vector antigen. We describe further possible advantages of viral vectors in achieving consistent antibody priming, enhanced antibody avidity, and cytophilic isotype skew. These data strengthen the evidence that tailored combinations of vaccine platforms can achieve desired combinations of immune responses, and further encourage the co-administration of antibody-inducing recombinant protein vaccines with T cell- and antibody-inducing recombinant viral vectors as one strategy that may achieve protective blood-stage malaria immunity in humans.

## 1 Introduction

The strong cellular immune responses induced by viral vectors have encouraged their clinical development as candidate vaccines against cancer and a number of intracellular pathogens, notably pre-erythrocytic infection by *Plasmodia*, *Mycobacterium tuberculosis* (TB) and HIV-1 [1]. Recombinant protein-in-adjuvant formulations have remained predominant in efforts to induce antibody responses against extracellular pathogens, including blood-stage malaria parasites [2]. Recently, replication-deficient viral-vectored vaccines encoding blood-stage malaria antigens have, like protein vaccines, proven protective in a rodent malaria model and induced promising *in vitro* activity in assays against *Plasmodium falciparum*[3–6].

Combined cellular and humoral responses may be desirable for maximal immune-mediated protective efficacy in a number of contexts, notably against malaria (both pre-erythrocytic and blood-stage) and HIV [6–9]. Despite the ongoing development of single antigen, single formulation vaccines many speculate that the first highly efficacious vaccine against *P. falciparum* malaria will require a multi-antigen, multi-stage, or multi-formulation product [7].

Multiple strategies using heterologous prime-boost combinations of DNA, viral vectored and protein vaccines have demonstrated capacity to induce combined antibody and cellular responses in the HIV field. Adenovirus prime–protein boost regimes induce greatly enhanced antibody immunogenicity compared to individual adenovirus or protein/adjuvant immunization, both in guinea pigs and primates [10,11]. Similarly, replication-competent-adenovirus prime–protein boost and triple platform DNA-Semliki Forest virus–orthopoxvirus combinations have proven immunogenic and protective in a macaque SIV model [12,13]. DNA–protein and DNA–poxvirus–protein candidate HIV-1 vaccine regimes have also entered phase I and II clinical trials [14–17], and a regime comprising a canarypox (ALVAC) prime and protein boost was recently reported to have induced partial protection against HIV-1 infection in a phase III clinical trial in Thailand [18]. Although this particular result requires further confirmation, it highlights the exciting potential of regimes combining viral vectors and recombinant proteins to induce protection against an immunologically challenging target.

In the malaria field, such approaches have been less thoroughly explored. Results of efforts to combine viral vectors encoding the pre-erythrocytic antigen circumsporozoite protein (CSP) with the leading CSP-based vaccine RTS,S (a non-vectored recombinant virus-like particle) have been mixed. A phase I/IIa clinical trial of modified vaccinia virus Ankara (MVA)-CSP prime with RTS,S boost did not enhance immunogenicity or protection beyond that achieved by RTS,S alone [19], in contrast to encouraging pre-clinical observations on the combination of MVA with hepatitis B surface antigen or *Plasmodium berghei* CSP proteins [20,21]. More recently, a macaque study using an adenovirus vectored-CSP prime and RTS,S boost significantly improved CD4^+^ T cell immunogenicity compared to the individual vaccines used alone, but did not enhance antibody responses above those seen with RTS,S [22].

Merozoite surface protein 1 (MSP1) is a leading candidate antigen for use in subunit vaccination against blood-stage *P. falciparum*, with numerous MSP1-based vaccines under development [2,23]. Vaccination with recombinant MSP1 can protect mice against *Plasmodium yoelii* challenge and *Aotus* monkeys against *P. falciparum*[24,25]. It is generally thought that the principal mechanism of MSP1-induced immunity is blockade of erythrocyte invasion by antibodies to the C-terminal MSP1_19_ moiety, though it has also been demonstrated that antibodies can arrest growth at a stage after erythrocyte invasion [26]. Antibodies against MSP1_19_ are responsible for a substantial proportion of the *in vitro* growth inhibitory activity of serum from individuals in *P. falciparum* endemic areas [27]. In addition to antibody, CD8^+^ T cell responses to MSP1 can provide partial protective efficacy against late liver-stage *P. yoelii* parasites [6,28], and CD4^+^ T cells specific to *P. yoelii* MSP1_33_ can confer protection against blood-stage infection when adoptively transferred into mice in the absence of antibodies [29]. Protection in humans against *P. falciparum* following whole-parasite immunization with both sporozoites and blood-stage parasites has been associated with T cell responses against blood-stage parasites, although drug persistence casts some doubt upon the results of the latter study [30–32]. In contrast, despite considerable effort and promising antibody induction, protein-based subunit vaccines have so far failed to induce substantial protection against blood-stage *P. falciparum*[2].

We were therefore interested to compare antibody induction by viral vectors head-to-head with protein-adjuvant vaccination, and to develop a regime which improved upon the antibody induction of our viral vectored regime while retaining its cellular immunogenicity. Adenovirus–MVA heterologous prime–boost using a PfMSP1 antigen insert is a leading viral vectored regime for antibody and T cell induction against this blood-stage *P. falciparum* antigen [3,5]. As a protein-adjuvant comparator, we used a *Pichia pastoris*-expressed recombinant PfMSP1_19_[33], adjuvanted by Montanide ISA720 (Seppic, France). Montanide ISA720 is a squalene-based water-in-oil emulsion which has been shown to be a potent adjuvant in both animal and human studies [34–37].

Here we describe and compare in detail the immunogenicity of PfMSP1 vaccines using a novel combination of three subunit vaccine platforms: simian adenovirus AdCh63 [5,38]; MVA; and recombinant protein in Montanide ISA720. We report that, when combined, these technologies can achieve simultaneous antibody and T cell responses which equal, or in some cases surpass, the best immune responses achieved with either technology alone. We describe in detail the responses induced, with data on antibody isotypes and avidity, splenic antibody secreting cell counts, T cell quality, and response longevity.

## 2 Materials and methods

### 2.1 Animals and immunizations

All procedures were performed in accordance with the terms of the UK Animals (Scientific Procedures) Act Project Licence and were approved by the University of Oxford Animal Care and Ethical Review Committee. 5–6 weeks old female BALB/c (H-2^d^) and C57BL/6 (H-2^b^) mice (Harlan Laboratories, Oxfordshire, UK) were anesthetized before immunization with medetomidine (Domitor, Pfizer) and ketamine (Ketaset, Fort Dodge) and revived subsequently with Antisedan reversal agent (Pfizer). All immunizations were administered intramuscularly (i.m.) unless otherwise specified, with vaccine divided equally into each musculus tibialis.

The creation of simian adenovirus 63 (AdCh63) and modified vaccinia virus Ankara (MVA) vectors encoding the PfM128 antigen is described elsewhere [5]. Briefly, this antigen is a bi-allelic fusion incorporating the MSP1_42_ antigen from the K1/Wellcome and 3D7/MAD20 *P. falciparum* strains fused in tandem alongside four blocks of conserved sequence from the remainder of the 3D7 strain MSP1 molecule (blocks 1, 3, 5 and 12). The MVA used in the current study differs from the previously published vector [3] in that it lacked the green fluorescent protein (GFP) marker. To generate the markerless MVA expressing PfM128, the antigen was cloned into a transient-dominant shuttle vector plasmid such that PfM128 was expressed from the vaccinia P7.5 promoter, and inserted into the TK locus of MVA. The plasmid also expresses a GFP marker [39]. This plasmid was transfected into chicken embryo fibroblast cells (CEFs) infected with MVA expressing red fluorescent protein (RFP), as previously described [3]. Recombinant MVAs were generated by homologous recombination between regions of homology at the TK locus of MVA and in the plasmid shuttle vector. Unstable intermediate recombinants expressing RFP and GFP were selected using a MoFlo cell-sorter (Beckman Coulter, USA) and plated out on CEFs. After 2–3 passages, further recombination between the repeated TK flanking regions results in either reversion to the starting virus (MVA–RFP) or formation of the markerless recombinant virus MVA-PfM128. White plaques (expressing neither RFP nor GFP) were picked and purified. Presence of the PfM128 antigen at the TK locus was confirmed by sequencing and PCR.

The protein vaccine used was mono-allelic Wellcome strain MSP1_19_ expressed in the yeast *P. pastoris* (kindly provided by A Holder, NIMR, London) [33]. The full sequence of this antigen is represented within the viral vector vaccines. Protein in endotoxin-free PBS was mixed manually in a syringe immediately prior to immunization with Montanide ISA720 adjuvant (SEPPIC, France), in the ratio 3:7 as previously described [40]. Where applicable, viral vectored vaccines were incorporated in the protein-PBS fraction of this mixture.

BALB/c mice were vaccinated at 8- or 14-week intervals with doses as follows (unless otherwise specified): 10^10^ virus particles (vp) for AdCh63; 10^7^ plaque forming units (pfu) for MVA; and 20 μg of protein. C57BL/6 mice were vaccinated at 8-week intervals with 10^8^ vp AdCh63, 10^6^ pfu MVA, or 5 μg protein. Blood was obtained for immunological studies using tail bleeds 2 weeks after each immunization and at later time points as described.

### 2.2 *Ex vivo* IFNγ and splenic antibody-secreting cell ELISPOT

*Ex vivo* IFNγ enzyme linked immunosorbent assays (ELISPOT) were performed as previously described [41], using peptides appropriate to the mouse strain as follows: either the overlapping peptides 90 and 91 (NKEKRDKFLSSYNYI and DKFLSSYNYIKDSID) which comprise the immunodominant CD8^+^ T cell epitope in PfMSP1_33_ (Wellcome allele) in BALB/c mice; or the PfMSP1_19_ (3D7 allele)-derived peptide 215 (TKPDSYPLFDGIFCS) recognised by CD8^+^ T cells from C57BL/6 mice [5].

Antigen-specific splenic antibody secreting cells (ASCs) were measured as previously described [42]. In brief, nitrocellulose bottomed 96-well Multiscreen HA filtration plates (Millipore, UK) were coated with 5 μg/ml *P. falciparum* MSP-1_19_ (Wellcome/FVO allele, expressed in *Pichia*) [33] and incubated overnight at 4 °C. Plates were washed twice with PBS and blocked for 1 h at 37 °C, 5% CO_2_ with D10 (MEM α-modification, 10% Fetal Calf Serum, 4 mM l-glutamine, 100 U/ml penicillin and 100 μg/ml streptomycin (all from Sigma, UK); and 50 μm 2-mercaptoethanol (Gibco)). 5 × 10^5^ splenocytes were plated onto the pre-coated ELISPOT plate per replicate well and serially diluted. Plates were incubated for 5 h at 37 °C, 5% CO_2_. Following incubation plates were washed twice with PBS and incubated overnight at 4 °C with biotinylated anti-mouse γ-chain specific IgG antibody (CALTAG, CA). Assays were developed using colour developing agents (Bio-Rad AP conjugate substrate kit) that were filtered through a 0.2 μm filter (Sartorius, UK). ELISPOT plates were counted using AID plate reader software (AID, Cadama Medical) and counts were visually confirmed. No spots were observed in control wells containing splenocytes but no coating antigen.

### 2.3 Intracellular cytokine staining

**Supplementary Figure 1:**
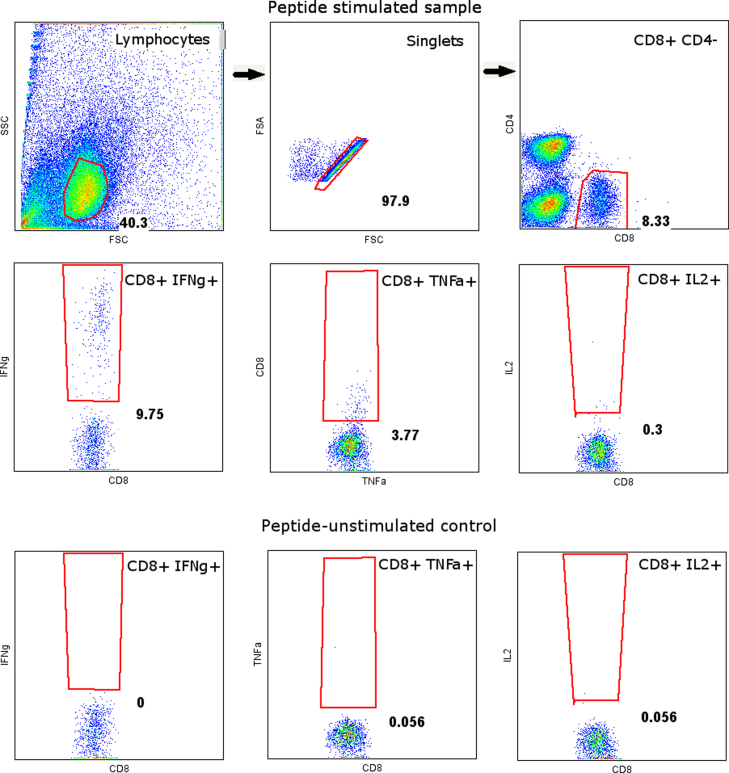
ICS gating strategy for a representative sample (A–M–P immunized, 2 weeks after final vaccination). The upper six panels illustrate responses in a peptide-stimulated well. The lower three panels are the peptide-unstimulated control for the same sample.

The percentage of peripheral blood and splenic CD8^+^ T cells expressing IFNγ, TNFα and IL-2 in response to 5 h stimulation with 5 μg/ml peptides 90 and 91 was assessed by intracellular cytokine staining as previously described [5]. Surface staining was with anti-CD8α PerCP-Cy5.5 and anti-CD4 Pacific Blue while intracellular staining was with anti-IFNγ APC, anti-TNFα FITC and anti-IL-2 PE (all supplied by eBioscience, UK). Cytokine production frequency in peptide-unstimulated control wells (which was typically <0.1%) was subtracted from the result in peptide-stimulated wells prior to further analysis. The gating strategy is illustrated in supplementary Figure 1.

### 2.4 Antibody responses—total IgG, isotypes and avidity

Total IgG and isotype ELISA were carried out as previously described using bacterially expressed GST-tagged PfMSP1_19_ (Wellcome/FVO allele) as the coating antigen [5].

Antibody avidity was assessed by sodium thiocyanate (NaSCN)-displacement ELISA [43]. Using previously measured total IgG ELISA titers, sera were individually diluted to a level calculated to give a titer of 1:300 and plated at 50 μl/well in 16 wells of a 96 well plate. Following incubation and washing, an ascending concentration of the chaotropic agent NaSCN was added down the plate (0–7 M NaSCN). Plates were incubated for 15 min at room temperature before washing and development as for total IgG. The intercept of the OD_405_ curve for each sample with the line of 50% reduction of the OD_405_ in the NaSCN-free well for each sample (i.e. the concentration of NaSCN required to reduce the OD_405_ to 50% of that without NaSCN) was used as a measure of avidity.

### 2.5 Statistical analysis

Statistical analysis was carried out using Prism 5 software (GraphPad, La Jolla, CA, USA). All ELISA titers were log_10_ transformed prior to analysis. Graphs indicate sample arithmetic means; error bars where present indicate 95% confidence intervals for the population arithmetic mean. One-way ANOVA was used for comparing normally distributed data with Bonferroni's multiple comparison post-test for comparison of specific groups; Kruskal–Wallis tests were used for comparison of non-normally distributed data with Dunn's multiple comparison post-test for comparison of specific groups. Two-way ANOVA was used for comparison of groups differing in two factors. Two-way repeat measures ANOVA was used for comparison of responses measured for different groups at different time points, after the exclusion of the small number of mice for which replicate data were not available at all time points. *P* < 0.05 was taken to be statistically significant throughout.

## 3 Results

### 3.1 Immunogenicity of two component regimes

**Fig. 1:**
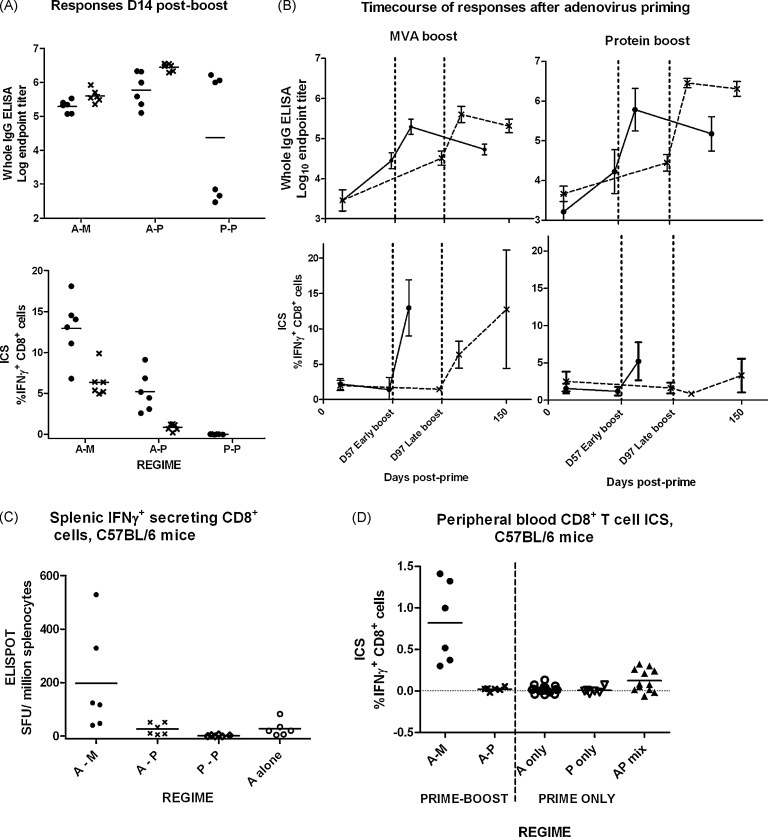
Responses induced by two-immunization regimes. In figures throughout this report, the abbreviations ‘A’, ‘M’ and ‘P’ are used in place of ‘AdCh63’, ‘MVA’ and ‘protein’, respectively. A dash is used to indicate separate sequential vaccinations whereas parentheses and a+ sign indicates mixed vaccinations–for example, ‘A–P’ indicates AdCh63 followed by protein, whereas ‘(A+P)’ indicates mixed adenovirus and protein given simultaneously at the same site. Unless otherwise stated, mice were BALB/c and doses were 10^10^ virus particles (vp) for AdCh63; 10^7^ plaque forming units (pfu) for MVA; and 20 μg for protein. In this and subsequent figures, all graphs plot individual values (symbols) and group mean (line). Error bars, where present, indicate 95% confidence interval for population mean. Throughout, group size *n* = 6 except where otherwise specified. Panel A: Comparison of total PfMSP1_19_ specific IgG and PfMSP1_33_ specific CD8^+^ T cell responses measured by ICS 14 days post-boost in BALB/c mice. P–P 97 day dose interval not done. Graph symbols indicate dose interval: (●) for 57 days; (×) for 97 days. Panel B: Illustration of timecourses of total IgG (upper two panels) and CD8^+^ T cell (lower two panels) responses in adenovirus-primed BALB/c mice receiving different boosting vaccines (MVA in left two panels; protein in right-hand two panels). In each panel, the two lines illustrate responses with different dosing intervals, with graph symbols as in panel A. Panel C: C57BL/6 mice were immunized for examination of splenic IFNγ secreting CD8^+^ T cell response to an MSP1_19_ epitope (peptide 215), assessed by ELISPOT for optimal sensitivity 14 days after vaccination. Doses were 10^8^ vp AdCh63, 10^6^ pfu MVA, and 5 μg protein. Panel D: C57BL/6 mice (*n* = 6–18 per group) were immunized as in Figure C, with the addition of a group receiving a single immunization with adenovirus–protein mixture. CD8^+^ T cell responses to peptide 215 were assessed by peripheral blood ICS 14 days after vaccination.

The experimental design provided replicate groups receiving AdCh63–MVA (A–M) and AdCh63–protein (A–P) sequential regimes at 57 day and 97 day intervals. Antibody and IFNγ^+^ CD8^+^ T cell responses induced by these regimes are illustrated in Fig. 1. These data were analyzed by two-way ANOVA, demonstrating that antibody responses 14 days post-boost were greater with the A–P regime than the A–M regime (Fig. 1A) (*P* < 0.0001), and greater with the 97 day interval than the 57 day interval (*P* = 0.0006). The antibody response induced by protein–protein (P–P) vaccination was markedly variable with three mice mounting high responses comparable to those receiving A–P immunization, and three very weakly responding mice (Fig. 1A and B). There was no significant difference between median antibody responses following protein–protein, adenovirus–MVA and adenovirus–protein regimes after a 57 day dose interval (*P* = 0.37 by Kruskal–Wallis test), but there was a clear increase in the variance of the response after two shot protein regimes compared to viral-vector containing regimes.

In contrast with the antibody results, greater percentages of IFNγ^+^ CD8^+^ T cells were detected by ICS 14 days after A–M immunization than A–P, and the 57 day dose interval was superior (*P* < 0.0001 for both comparisons) (Fig. 1A and B). Clear boosting of CD8^+^ T cell responses by MVA was evident at both dose intervals. As expected, given the lack of the CD8^+^ T cell epitope in the MSP1_19_ protein sequence in BALB/c mice [5], CD8^+^ T cell responses were not detectable following P–P vaccination. Additional experiments in C57BL/6 mice (in which a CD8^+^ T cell epitope is present in the MSP1_19_ protein [5]) confirmed that, in contrast to the A–M regime, P–P vaccination did not induce a CD8^+^ T cell response detectable by IFNγ splenic ELISPOT or peripheral blood ICS, and that CD8^+^ T cell responses were unaltered by A–P immunization as compared to adenovirus priming alone (Fig. 1C and D). CD8^+^ T cell responses after A–P immunization of either mouse strain thus presumably represent the contracting or effector memory CD8^+^ T cell response induced by the adenovirus.

### 3.2 Immunogenicity of three-component sequential regimes

**Fig. 2:**
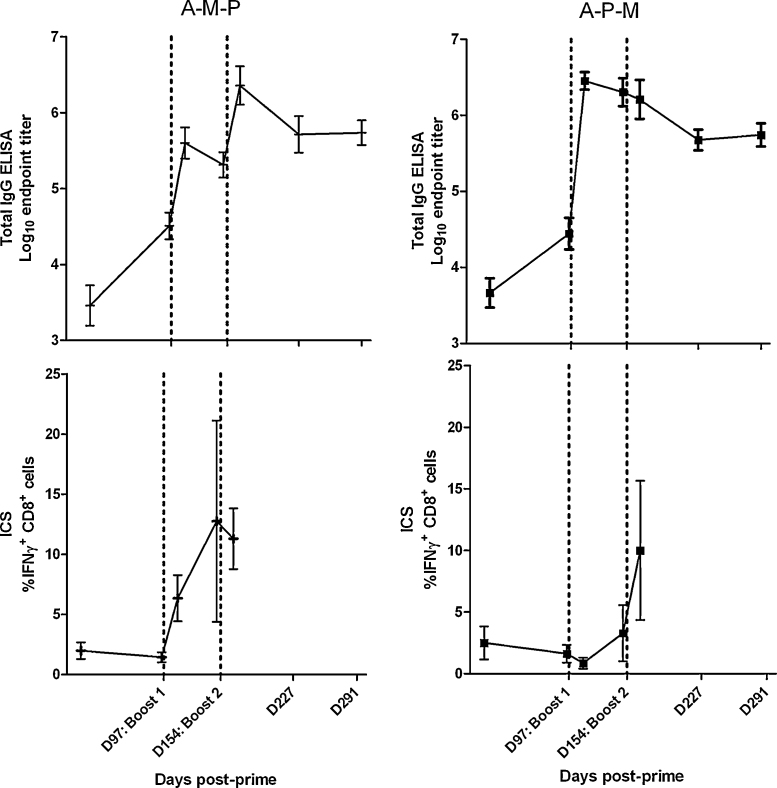
Kinetics of responses to A–M–P (panel A) and A–P–M (panel B) three-stage immunization regimes in BALB/c mice. Regimes as described in legend to Fig. 1. All mice were primed at day 0, with subsequent boosts on days 97 and 154, as indicated by dotted vertical lines. Upper panels depict PfMSP1_19_ specific total IgG responses assessed by ELISA. Lower panels depict PfMSP1_33_ specific IFNγ^+^ CD8^+^ T cell responses assessed by ICS. Late time point T cell responses assayed by ELISPOT are presented in Fig. 5B.

**Fig. 3:**
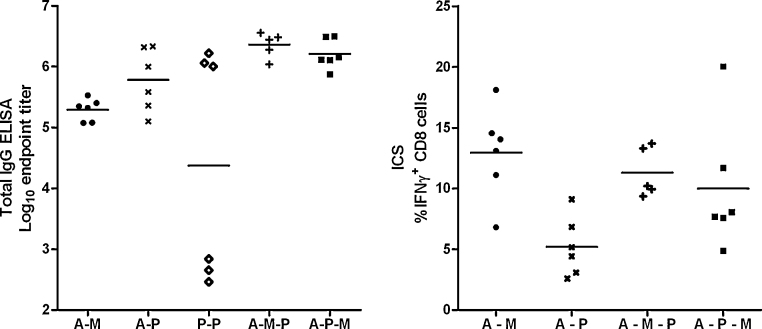
Comparison of responses of BALB/c mice to three component and two component sequential regimes. Dosing was as described in legend to Fig. 1; *n* = 6/group. Mice receiving three vaccinations were primed at day 0, with subsequent boosts on days 97 and 154 (the same mice are depicted in Fig. 1 with 97 day interval and in Fig. 2). Mice receiving two vaccinations received these on days 97 and 154. This permitted all results depicted in this figure to be obtained from synchronous assays 14 days after final vaccination. All subsequent figures use data from the same groups of mice, with the exception of dose interval comparisons in Fig. 8 and low dose data in supplementary Figure 2. Panel A: Total IgG responses assessed by ELISA. Panel B: IFNγ^+^ CD8^+^ T cell responses assessed by ICS.

We subsequently compared the immunogenicity of three-component sequential adenovirus–MVA–protein (A–M–P) and adenovirus–protein–MVA (A–P–M) regimes to two-component regimes (Figs. 2 and 3). The kinetics of the responses induced by these regimes were markedly different. We found that addition of protein to adenovirus–MVA (A–M–P) was able to boost antibody but not CD8^+^ T cell responses (again as would be predicted due to lack of the T cell epitope in this protein) (Fig. 2A), while addition of MVA to adenovirus–protein (A–P–M) boosted CD8^+^ T cell responses but not antibody titer (Fig. 2B). Total IgG responses to A–M–P and A–P–M were significantly higher than those to A–M (*P* < 0.05 by ANOVA with Bonferroni post-test), with no significant differences between the responses to A–M–P, A–P–M and A–P (*P* > 0.05, Fig. 3A). There were no statistically significant differences in CD8^+^ T cell responses between A–M–P, A–P–M and A–M regimes (*P* > 0.05 by ANOVA with Bonferroni post-test, Fig. 3B). In general, any two- or three-component regime including AdCh63 and MVA induced maximal CD8^+^ T cell responses as measured in the blood. Conversely, maximal IgG responses were elicited by any regime including AdCh63 and protein.

### 3.3 Regimes mixing viral-vectored and protein-adjuvant vaccines

**Fig. 4:**
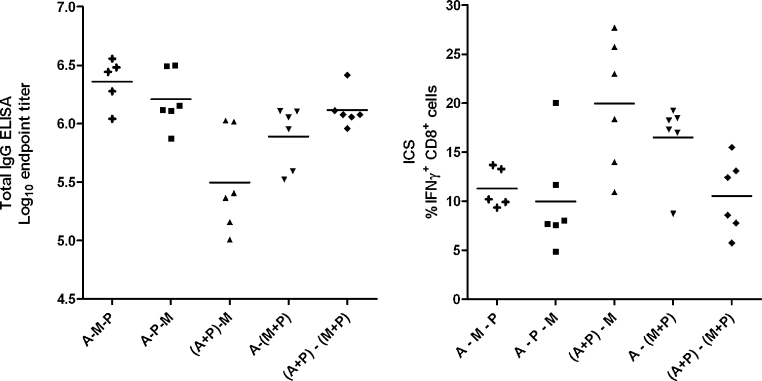
Comparison of responses of BALB/c mice to three component sequential and two-stage mixed-component regimes. Vaccination regimes were as described in legend to Fig. 3 with doses as in legend to Fig. 1. This permitted all results depicted in this figure to be obtained from synchronous assays 14 days after final vaccination. Data for groups A–M–P and A–P–M are as displayed in Fig. 3, repeated here for clarity. Panel A: Total IgG responses assessed by ELISA. Panel B: IFNγ^+^ CD8^+^ T cell responses assessed by ICS.

We continued to investigate whether the advantages of three-component regimes could be achieved in a simplified two-stage regime, by mixing protein and adjuvant with one or both viral vector components (Fig. 4A and B). We found that there was no significant difference by Kruskal–Wallis test between the three-immunization regimes and a two-immunization regime mixing protein and Montanide ISA720 with both adenovirus prime and MVA boost. Interestingly, there was a small but statistically significant increase in CD8^+^ T cell responses and decrease in antibody responses with the (A+P)–M regime relative to A–P–M (*P* < 0.05, ANOVA with Bonferroni post-test). Antibody responses tended to be highest with the three component regimes, or when protein-adjuvant was co-administered with both viral vectors. Interestingly, in C57BL/6 mice, (A+P) priming induced modestly but significantly higher CD8^+^ T cell responses than adenovirus alone (Fig. 1D, *P* = 0.04, Mann–Whitney test).

Thus a simplified two-shot immunization regime appears highly immunogenic and mixing of the viral vectors with protein and adjuvant did not appear to affect vector potency, a result which may encourage development of further strategies combining vectors with protein and adjuvant, including homologous vector–protein prime–boost immunization regimes.

### 3.4 Longevity of responses

**Fig. 5:**
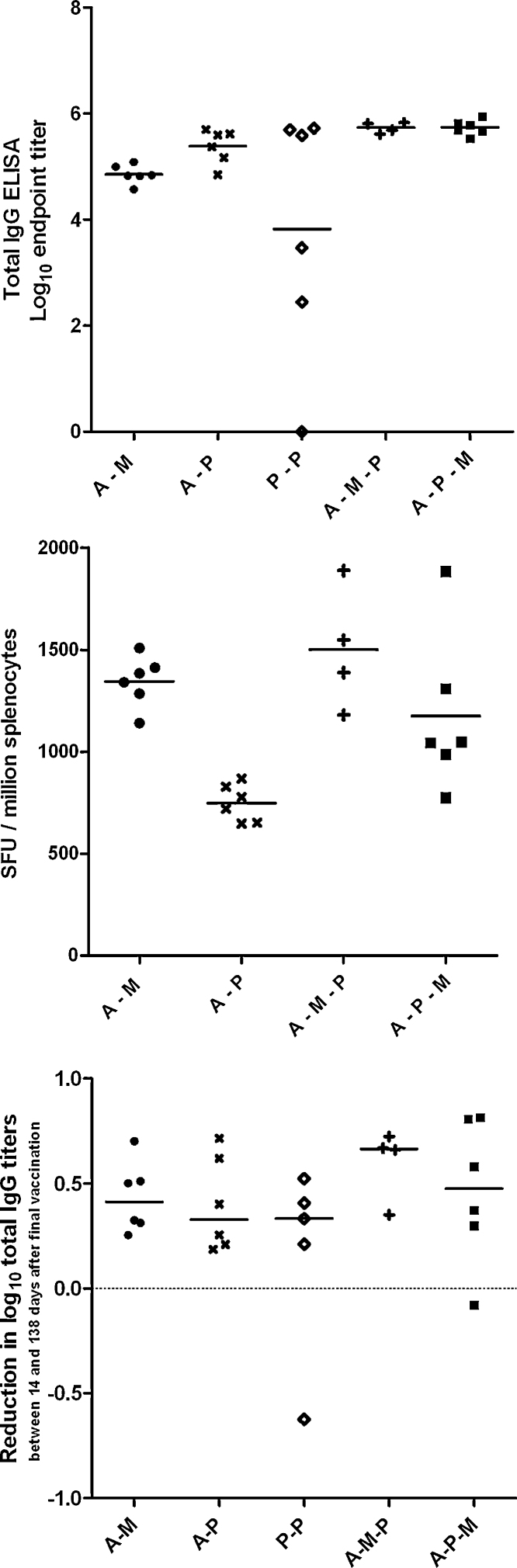
Persistent immune responses 20 weeks after final vaccination in BALB/c mice. Mice and regimes were as described in legend to Fig. 3. Panel A: Total IgG responses assessed by ELISA. Panel B: IFNγ secreting CD8^+^ T cell responses assessed by *ex vivo* splenic ELISPOT. SFU = spot forming units. Panel C: Comparison of reduction in log IgG titer between 14 and 137 days after final vaccination with different regimes.

Serum antibody and splenic T cell responses were assayed by ELISA and IFNγ ELISPOT 138 days after final vaccination for selected groups of mice (Fig. 2 D291 time point and Fig. 5). Antibody responses to A–M–P and A–P–M remained significantly higher than those for A–M (*P* < 0.05 for both comparisons by Kruskal–Wallis test with Dunn's multiple comparison post-test), while CD8^+^ T cell responses following A–M–P and A–M remained greater than those for A–P (*P* < 0.01 and *P* < 0.05 respectively by the same method). There was a mean drop of 0.4 log units in ELISA titer between 14 and 138 days after final vaccination, with no significant difference in this rate of decline between groups (Fig. 5C, *P* = 0.37 by Kruskal–Wallis test). Thus, as was the case with early post-vaccination responses, maximal long-lived IgG responses were detected with any regime including AdCh63 and protein, while any regime including AdCh63 and MVA induced maximal long-lived CD8^+^ T cell responses in the spleen.

### 3.5 Immunization routes and doses

**Fig. 6:**
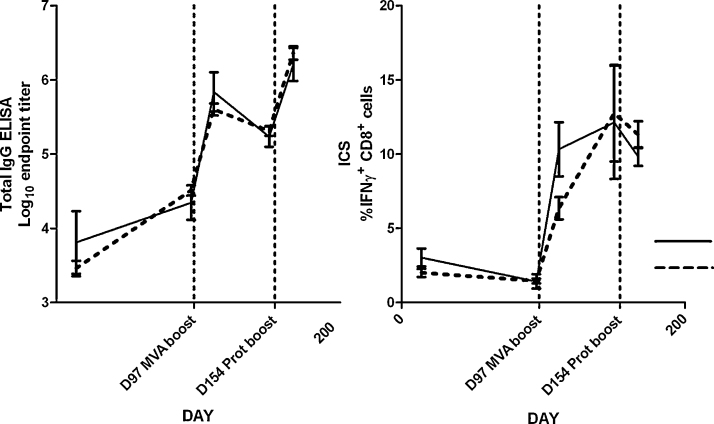
Comparison of intradermal and intramuscular injection route for AdCh63 and MVA. BALB/c mice were immunized with adenovirus 1 × 10^10^ vp and MVA 1 × 10^7^ pfu, either receiving both vectors i.d. or both vectors i.m. All mice received a third vaccination with 20 μg protein i.m. Timing was as described in legend to Fig. 3. Panel A: Total IgG responses assessed by ELISA. Panel B: IFNγ^+^ CD8^+^ T cell responses assessed by ICS.

We also compared the antibody and CD8^+^ T cell responses of six mice receiving the A–M–P regime entirely intramuscularly versus six mice receiving the viral-vector components intradermally (i.d.) (Fig. 6). There was no significant difference by t-test between the two groups’ log ELISA titer (*P* = 0.26) or % IFNγ^+^ CD8^+^ T cells (*P* = 0.20) 14 days after final vaccination, nor was a difference found between groups for either ELISA or CD8^+^ T cell responses by repeat measures ANOVA taking into account all time points up to 14 days after final vaccination.

**Supplementary Figure 2:**
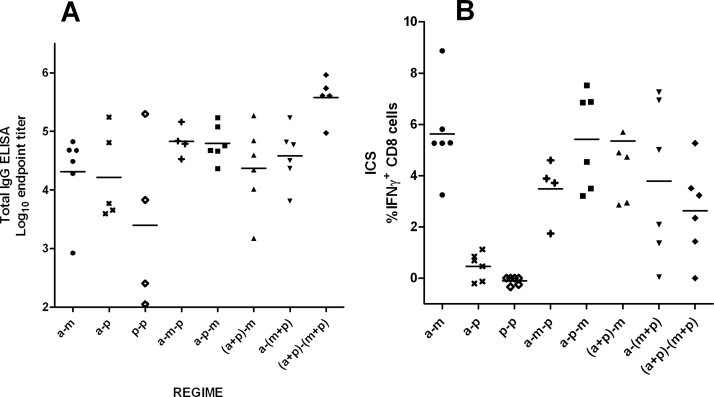
Comparison of immunogenicity of various low-dose vaccination regimes. BALB/c mice received various combinations of AdCh63 PfM128 (‘a’; 1 × 10^8^ vp i.m.), MVA PfM128 (‘m’; 1 × 10^6^ pfu i.m.); and PfMSP1_19_ protein (‘p’; 5 μg in Montanide ISA720). Vaccination regimes are indicated in *x*-axis labels. Panel A: Total IgG responses assessed by ELISA. Panel B: IFNγ^+^ CD8^+^ T cell responses assessed by ICS.

In parallel, we had conducted the same experiments at lower vaccine doses (10^8^ vp AdCh63, 10^6^ pfu MVA, and 5 μg protein at 8-week intervals) in BALB/c mice, in case a ‘ceiling’ or maximum dose–response effect prevented us observing differences between the higher dose regimes used in the previous experiments (supplementary Figure 2A and B). Importantly, similar patterns to those previously observed were apparent from the lower dose experiment. As expected all antibody and T cell responses were substantially weaker when using lower vaccine doses. Responses to protein–protein vaccination were markedly more variable than responses to adenovirus-containing regimes. At these lower doses, addition of protein did not enhance the antibody immunogenicity of viral vector regimes, with no significant differences in ELISA titers following A–M, A–P, A–M–P or A–P–M vaccination. T cell responses were again substantially higher in the A–M, A–M–P and A–P–M groups than in the A–P group. As before, the (A+P)–M, A–(M+P) and (A+P)–(M+P) two-stage regimes mixing viral and protein vaccines produced results similar to three-stage vaccination, with a trend towards higher antibody but lower CD8^+^ T cell responses in the group receiving (A+P)–(M+P). Thus despite the clearly sub-maximal responses achieved in these animals (in particular with the protein only vaccination), regimes incorporating adenovirus and MVA again appeared to result in more consistent combined antibody and CD8^+^ T cell responses to the antigen.

### 3.6 Antibody isotypes

**Fig. 7:**
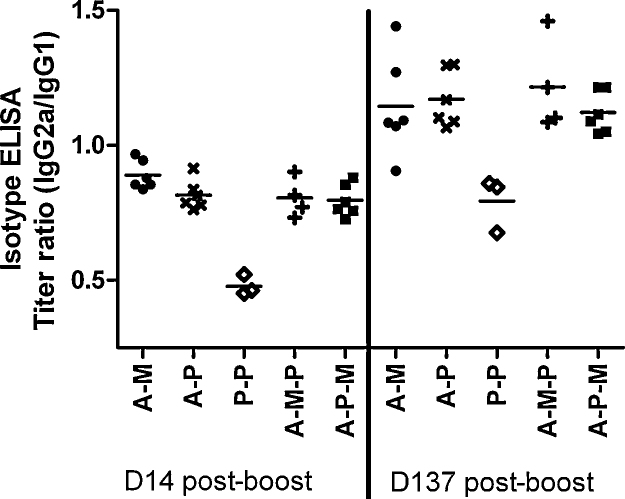
Titers of IgG1 and IgG2a were measured separately by isotype-specific ELISA, using sera taken from mice which had received five different vaccination regimes at two time points (14 and 137 days after final vaccination). Vaccination regimes were as described in legend to Fig. 3 with doses as in legend to Fig. 1. Data are plotted as ratio of log titer of IgG2a/IgG1.

To further characterize the immune responses to the various vaccine modalities, we performed IgG isotype ELISAs. It was not possible to measure isotype-specific titers for the three P–P immunized mice with low total IgG ELISA titers. Bearing in mind this limitation, viral-vector-containing regimes induced a significantly greater ratio of IgG2a to IgG1 than was present in the high-total-titer P–P immunized mice, and that the IgG2a/IgG1 ratio was higher for all groups 137 days rather than 14 days after the final vaccination, corresponding to better maintenance of the titer of IgG2a than IgG1 over time (Fig. 7; *P* < 0.001 for both comparisons by repeated measures two-way ANOVA with Bonferroni's post-test). There was no interaction of time and regime (i.e. no inter-regime differences in the rate of change of the IgG isotype balance over time).

### 3.7 Antibody avidity

**Fig. 8:**
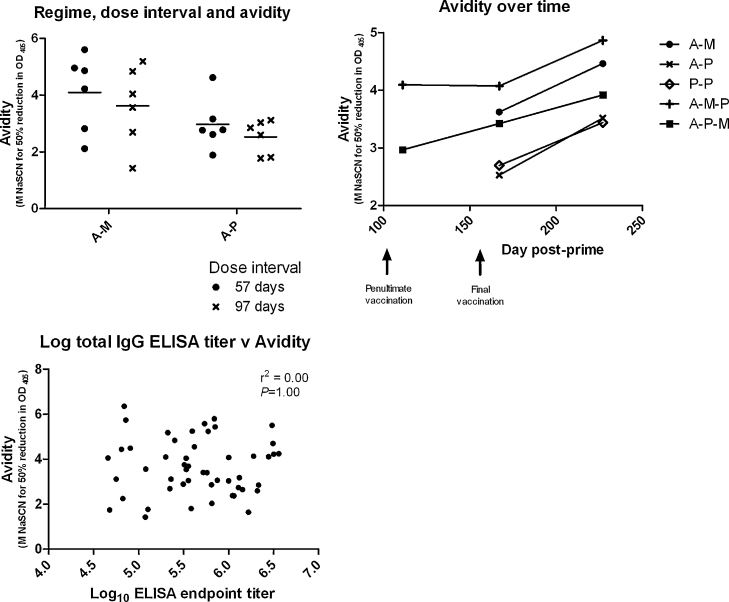
Antibody avidity was measured as the concentration of NaSCN required to reduce OD_405_ by 50% relative to sample OD_405_ without NaSCN. Vaccination regimes were as described in legend to Fig. 3 with doses as in legend to Fig. 1. Panel A: Avidity 2 weeks after boosting in mice receiving A–M and A–P regimes at 57 day and 97 day dose intervals. Individual values (symbols) and mean (line) are plotted for each group. Panel B: Comparison of avidity between regimes and over time. Arithmetic mean values are plotted for each group. Panel C: Correlation between total IgG ELISA titer and avidity: measurements of total IgG and avidity are plotted for each individual mouse.

We continued to investigate the responses to the various regimes by measuring antibody avidity using NaSCN antibody-displacement ELISA for selected groups and time points (Fig. 8A–C). Among mice receiving A–M and A–P regimes, we observed that mice receiving A–M had higher antibody avidity 14 days post-boost than those receiving A–P, without any significant difference between 57 day and 97 day dose interval (Fig. 8A; *P* = 0.024 for regime comparison, *P* = 0.33 for comparison dose interval by two-way ANOVA). Looking more widely at mice receiving A–M–P, A–P–M, A–M, A–P and P–P regimes, it was apparent that there was a trend for higher avidity in mice receiving any regime including both viral vectors (A and M) than in those receiving only A–P or P–P (Fig. 8B). When analyzed by two-way repeat measures ANOVA, this trend did not reach statistical significance (*P* = 0.32) without pooling of replicate groups (described above for A–P and A–M), though there was a significant increase in avidity over time after final vaccination across all groups (*P* < 0.0001). There was no correlation between total IgG ELISA titer and avidity, either when data from all time points were combined (Fig. 8C, *r*^2^ = 0.00, *P* = 1.00 by linear regression) or where each time point was analyzed separately (data not shown). Thus antibody avidity and total IgG ELISA titer appear to vary independently, and avidity appears to rise over time post-boost and with MVA-containing regimes.

### 3.8 Splenic antibody secreting cells

**Fig. 9:**
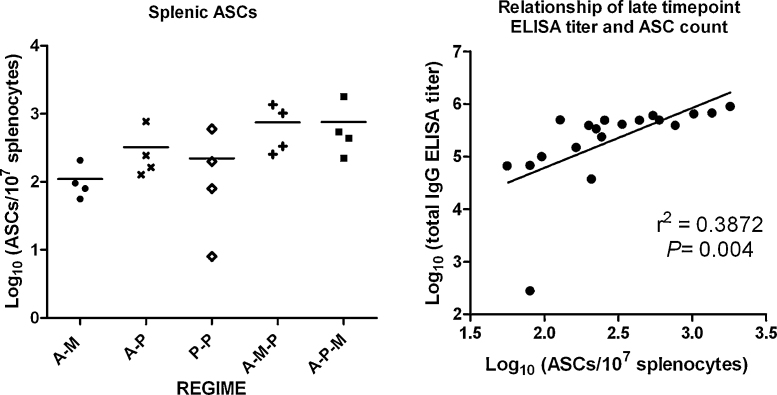
Antigen-specific antibody secreting cells (ASCs) were measured by *ex vivo* uncultured ASC ELISPOT 137 days after final vaccination in a random four mice from five groups receiving different vaccination regimes. Vaccination regimes were as described in legend to Fig. 3 with doses as in legend to Fig. 1. Panel A: Comparison of ASC counts between regimes. Individual values (symbols) and median (line) are plotted for each group. Panel B: Relationship of log transformed ASC count and ELISA titer 137 days after final vaccination. Line is best-fit linear regression line.

At the conclusion of the experiment (138 days after final vaccination), mice were sacrificed and antigen-specific antibody secreting cells (ASCs) in the spleens of four mice from each group were counted using an *ex vivo* assay without a proliferative culture step (Fig. 9). This non-cultured assay at such a late time point would be expected to detect the presence of long-lived plasma cells. Log transformed ASC counts differed between groups (*P* = 0.04 by Kruskal–Wallis test) with a trend towards the highest ASC counts in groups receiving three component regimes (A–M–P and A–P–M), and the lowest ASC count in mice receiving A–M. Differences between individual groups however did not reach statistical significance after correcting for multiple comparisons using Dunn's post-test. There was a reasonable linear correlation between log transformed ASC counts and log transformed total IgG ELISA titers, present using either peak ELISA titer 14 days after final vaccination (data not shown), or late ELISA titer 138 days after final vaccination (Fig. 9B, for late time point, *r*^2^ = 0.39, *P* = 0.004).

### 3.9 T cell functionality

**Fig. 10:**
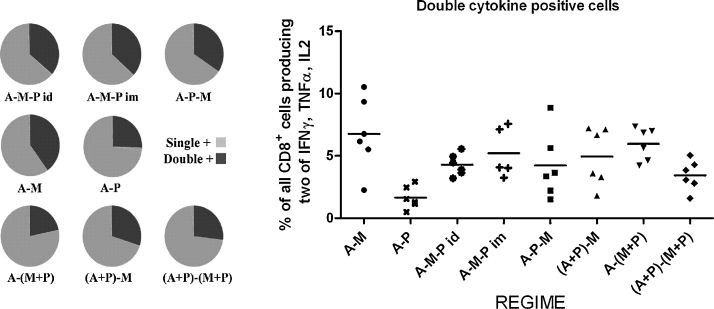
Intracellular cytokine staining allowed detection of cells producing combinations of IFNγ, TNFα and IL-2. Vaccination regimes were as described in legend to Fig. 3 with doses as in legend to Fig. 1. Gating strategy is illustrated in supplementary Figure 1. Panel A: Proportions of CD8^+^ T cells positive for at least one cytokine (usually IFNγ) or positive for a second cytokine (usually TNFα) 2 weeks after final vaccination. Produced using SPICE software (M. Roederer, NIH, USA). Panel B: Proportions of all CD8^+^ T cells which were positive for two cytokines.

The ICS antibody panel stained for IFNγ, TNFα and IL-2, thus allowing quantification of single, double and triple cytokine positive antigen-specific CD8^+^ T cells in the blood at the time points assayed. Results 2 weeks after final vaccination are displayed in Fig. 10. Given the lack of a CD8^+^ T cell epitope in the protein vaccine, the A–P group can be viewed as an unboosted control. The majority of T cells positive for a single cytokine were IFNγ^+^. Those positive for a second cytokine were mostly IFNγ^+^ TNFα^+^, in accordance with previous observations using viral-vector *P. yoelii* MSP1_42_ vaccines [6]. Few cells expressing IL-2 were observed with any regime. Comparing the various three-stage and two-stage regimes including both adenovirus and MVA, although there was some variation between regimes in the proportion of double cytokine positive cells relative to single positive cells (Fig. 10A), there was no difference in the proportion of double cytokine positive cells as a percentage of all CD8^+^ T cells (Fig. 10B) (*P* = 0.13 by ANOVA). Thus encouragingly, admixing viral vectors with protein-adjuvant did not affect either T cell quantity (Fig. 4B) or functional “quality”, demonstrating the potential at least in mice for these subunit vaccine platforms to be combined and administered using a single formulation.

## 4 Discussion

Adenoviral prime–MVA boost regimes induce antibody and CD8^+^ T cell responses equivalent or superior to a range of heterologous and homologous adenovirus-only two-stage regimes[5], making this immunization approach the current ‘gold-standard’ among adeno- and pox-viral vectored regimes. This study primarily sought to assess whether the antibody immunogenicity of our existing A–M PfMSP1 regime could be enhanced by the addition of a protein-adjuvant vaccine component, and has demonstrated that an encouraging combination of cellular and humoral responses can be achieved by this three-platform strategy.

The protein available to us – a *Pichia* produced, sequence-unmodified PfMSP1_19_ originally used in an NMR structural study – is likely to be conformationally accurate [33]. Good correlations between anti-PfMSP1_19_ ELISA titer and IgG-mediated *in vitro* growth inhibitory activity (GIA) against *P. falciparum* strains have previously been demonstrated both for our viral vectored vaccines and for a range of protein PfMSP1_19_ vaccines [5,44]. Direct GIA measurement was not possible with the small quantities of mouse serum available in this study.

As the protein antigen used here was only a portion of the viral-vector antigen, caution is necessary in the interpretation of our results. Although the use of BALB/c mice facilitated the investigation of antibody responses, which was our primary aim, some of the studies undertaken here could have benefited from detectable T cell responses against the MSP1_19_ moiety, which is small and poorly processed [45]. In future studies PfMSP1_42_ might be preferable as a protein antigen due to the known induction of T cell responses against MSP1_33_ epitopes in *P. yoelii* and *P. falciparum* as well as against PfMSP1_33_ in humans [5,6,46].

Despite this, our results clearly show that protein did not prime or boost appreciable CD8^+^ T cell responses in C57BL/6 mice in which a CD8^+^ T cell epitope is present in PfMSP1_19_. However, we have not yet fully investigated the potential effects of viral vector/protein-adjuvant mixing on CD8^+^ T cell responses when there is a CD8^+^ T cell epitope in a larger protein antigen that is less refractory to antigen processing. There is a possibility that CD4^+^ T cell responses at sub-detectable levels to epitopes present in the viral vector antigen but absent from the protein antigen may have contributed to the reliability of the viral vector priming, although the superior reliability of viral vector priming does not seem to be unique to this antigen (de Cassan et al., unpublished observations).

### 4.1 Antibody immunogenicity of triple platform regimes

Our results demonstrate that adenovirus is a highly reliable primer of antibody and CD8^+^ T cell responses. All 48 mice primed with 10^10^ vp of adenovirus had detectable antibody responses following a single vaccination, which were reliably boosted by MVA or protein. In contrast, recombinant protein vaccines require multiple doses to achieve consistently high antibody titers: five doses of *P. yoelii* MSP1_19_ in Freund's adjuvant are required for high and protective antibody titres [24] and three doses of RTS,S are required to achieve optimal titres in humans [47]. Although some mice receiving P–P in this study achieved high antibody titers, there was considerable variation within this regime.

Once responses were primed by adenovirus, protein appeared to be the optimal platform for boosting antibody responses with antibody titers after A–P exceeding those following A–M. Three-component regimes could also achieve simultaneous antibody and antigen-specific CD8^+^ T cell responses which equalled both antibody induction by adenovirus–protein and CD8^+^ T cell induction by A–M—hitherto the best regimes available. This pattern remained unchanged at time points up to 138 days after the final vaccination. Virus like particles (VLPs) are a fourth clinically relevant vaccine platform, noted for their ability to induce strong antibody responses. Adenovirus–MVA–VLP combinations may have potential to improve further upon the antibody results achieved here, while maintaining or enhancing viral-vector induced CD4^+^ and CD8^+^ T cell responses.

In the absence of a vaccine which protects humans against blood-stage *P. falciparum*, it is not yet fully understood which attributes of an antibody response are protective. In animal challenge models the induction of high antibody concentrations seems to be the principal predictor of MSP1 and AMA1 vaccine-mediated protection [48,49]. Most published work in the field simply uses ELISA titer as a quantitative readout of antibody induction. There are a further four quantitative properties of the vaccine-induced antibody response which we believe to be of interest: isotype balance; antibody avidity; rate of decline of ELISA titer; and recall response to re-exposure to antigen. The current results demonstrate significant differences between the viral vectored PfMSP1-based vaccines and the protein-adjuvant PfMSP1_19_ vaccine in some of these attributes. These may be due to differences between viral vector and recombinant protein delivery platforms or differences in the processing and inherent antigenicity of the differently sized antigens.

There are conflicting data regarding the importance of Fc-dependent functions of Th1-type cytophilic antibody subclasses (human IgG1 and IgG3; murine IgG2a and IgG2b) in protection against blood-stage malaria, and the impact of Th1 cytokines and IgG isotype on protective efficacy [50–53]. However, the balance of evidence now appears to support an important role of antibody–Fc receptor interactions in the response to the MSP1 and MSP3 antigens [54,55], and antibody-dependent respiratory burst production by neutrophils has been shown to correlate with protection against clinical malaria [56]. Protein-adjuvant vaccines often elicit relatively Th2 skewed responses with little murine IgG2a/b production [57]. Thus the significant enhancement of IgG2a production we observed with viral vectors here may be of protective value, particularly if it generalizes to other antigens postulated to induce Fc-dependent responses.

Antibody avidity has not been demonstrated to correlate with protection against blood-stage malaria and has in fact been predicted to be unimportant in response to merozoite antigens [48,58]. The relationship between avidity and protection in other diseases is complex and variable, but avidity has been observed to be associated with protection against respiratory syncytial virus, HIV-1 and anthrax [59–62]. The finding of enhanced avidity with A–M and related regimes compared to protein vaccination therefore merits further study and may be of interest beyond the malaria field.

There was strikingly little variation in the rate of decline of total IgG ELISA titer over the prolonged period of follow-up after vaccination. It would therefore seem that peak ELISA titer is an adequate predictor of antibody concentration at a later time point. The presence of a correlation between splenic ASC counts and ELISA titer at both early and late time points supports this. The reliable priming of antibody responses by adenovirus prior to subsequent boosting by MVA or protein strongly suggests that adenovirus containing regimes reliably generate memory B cell responses. It remains to be seen whether the different vaccine modalities investigated here induce memory B cell/antigen-recall responses that vary independently of peak antibody titer/overall regime immunogenicity.

It is interesting to note that in our previous studies, the viral vector PfMSP1-based antigen failed to induce detectable antigen-specific CD4^+^ T cell responses in BALB/c mice, even though viral vectored regimes can induce measurable CD4^+^ T cell responses against other antigens [5,6,63]. This would appear at odds with our finding of a reliably primed and boosted, avid, IgG2a skewed response to A–M-containing regimes: a response which bears the hallmarks of a Th1 response to a ‘T-dependent’ antigen bearing CD4^+^ T cell epitopes. Quite possibly, such helper T cell responses were simply below the limit of detection of the ICS assay, or these cells secreted cytokines other than IFNγ, TNFα and IL-2. Alternatively, recent evidence shows that, in mice, IFNα- or IFNγ-activated DCs can drive T-independent immunoglobulin class-switching with either a Th1 or Th2 skew, and that T-independent type-2 antigens can induce long-lived cells capable of mounting a secondary recall response [64,65]. It is therefore possible that adjuvants (and viral vectors) may be able to influence class-switching in a CD4^+^ T cell-independent manner.

### 4.2 Optimised regimes for clinical trials

Previous studies have demonstrated that addition of protein to DNA or MVA vaccines can enhance both antibody and T cell immunogenicity (but without investigating mixtures of recombinant adenoviruses and protein) [20]. The current study is not directly comparable due to its use of a different antigen and T cell assay (ICS), but given that adenovirus–MVA prime–boost generally results in higher antibody and T cell responses than DNA–MVA vaccination [66,67], it seems likely that the three-platform regimes reported here would out-perform combinations of DNA, MVA and protein.

Increasing the complexity of a viral vector vaccine regime by addition of protein and adjuvant components would clearly have cost implications, but these may be offset if fewer vaccine doses are required due to enhanced immunity induced. It has been reported elsewhere that the aluminium-based adjuvant Adjuphos can enhance responses from an AdHu35 vectored vaccine [68]. Our results with a two-shot regime co-administering viral vector and protein-Montanide ISA720 vaccines demonstrate that such admixture need not adversely affect the immunogenicity of either component, and that increasing the breadth of an immune response need not come at the cost of a regime which requires logistically difficult multiple immunizations. The observation in C57BL/6 mice that (A+P) priming may enhance CD8^+^ T cell responses above those induced by adenovirus alone merits further study.

The applicability of this triple-platform approach to human vaccination requires further investigation. Optimal doses in different species are usually not simply proportionate to body weight. We have used relatively high mouse doses to explore what are likely to be the maximal responses obtainable with each vaccine platform. Although it is possible that protein doses larger than the 20 μg used here could result in more reliable priming (and doses up to 160 μg have been used in human trials [69]), 20 μg is commonly used for mouse studies in this field [24]. It is worth noting that mean antibody titers in mice receiving a low-dose A–P regime were comparable to those in mice receiving a high-dose 20 μg protein-only P–P regime (Fig. 1A and supplementary Figure 2), although titers were more variable in the latter group. Regimes combining viral vectors and protein may therefore achieve a protein dose-sparing effect (high-dose viral vector, low-dose protein may prove optimal).

Overall this study has provided a detailed description of the immunogenicity of adenovirus–poxvirus–protein triple platform vaccination regimes, which we believe are likely to offer significant improvement upon the already promising results of previous vector–protein combinations. We have therefore progressed to test these results with other antigens and in larger animal species. It will also be important to test the protective efficacy of such regimes, either using rodent malaria antigens or possibly using *P. berghei* parasites transgenic for PfMSP1_19_[70,71]. There are strong animal and human data to support the importance of antibody concentration, CD4^+^ and CD8^+^ T cell responses in protection against malaria [6,29–31,72]. Given the failure to achieve protection of humans with PfMSP1-based protein vaccines to date [2], we propose that experimental vaccines should aim for maximal breadth of antibody and T cell responses; breadth which we have demonstrated can be achieved, along with potentially beneficial changes in avidity and isotype, by three component regimes including adenovirus, MVA and protein. Our favoured regime for a clinical trial of this approach would be either adenovirus or adenovirus/protein mix prime, followed by MVA/protein mix boost (with the choice of prime depending on whether protein dose-sparing was a consideration). These approaches require only a brief and practical two-shot vaccination regime, while achieving optimal T cell and antibody responses simultaneously.
